# Electrotransfer of Plasmid DNA Encoding an Anti-Mouse Endoglin (CD105) shRNA to B16 Melanoma Tumors with Low and High Metastatic Potential Results in Pronounced Anti-Tumor Effects

**DOI:** 10.3390/cancers8010003

**Published:** 2015-12-24

**Authors:** Tanja Dolinsek, Gregor Sersa, Lara Prosen, Masa Bosnjak, Monika Stimac, Urska Razborsek, Maja Cemazar

**Affiliations:** 1 Department of Experimental Oncology, Institute of Oncology Ljubljana, Zaloska 2, SI-1000 Ljubljana, Slovenia; tdolinsek@onko-i.si (T.D.); gsersa@onko-i.si (G.S.); laraprosen@yahoo.com (L.P.); mbosnjak@onko-i.si (M.B.); mstimac@onko-i.si (M.S.); urska09@gmail.com (U.R.); 2 Faculty of Health Sciences, University of Primorska, Polje 42, SI-6310 Izola, Slovenia

**Keywords:** endoglin, melanoma, gene electrotransfer, electroporation, shRNA, mice

## Abstract

Endoglin overexpression is associated with highly proliferative tumor endothelium and also with some tumors, including melanoma. Its targeting has anti-tumor effectiveness, which can also be obtained by RNA interference. The aim of our study was to explore the anti-tumor effectiveness of endoglin silencing by electrotransfer of plasmid DNA encoding short hairpin RNA against endoglin in two murine B16 melanoma variants with different metastatic potential on cells, spheroids and subcutaneous tumors in mice. The results demonstrate that endoglin silencing with gene electrotransfer reduces the proliferation, survival and migration of melanoma cells and also has anti-tumor effectiveness, as the therapy resulted in a high percentage of tumor cures (23% and 58% on B16F1 and B16F10 tumors, respectively). The effectiveness of the therapy correlated with endoglin expression in melanoma cells; *in vitro* the effects were more pronounced in B16F1 cells, which express more endoglin than B16F10. However, the opposite was observed *in vivo* in tumors, where there was a higher expression of endoglin and better anti-tumor effectiveness in the B16F10 tumor. In conclusion, targeting endoglin for the treatment of melanoma seems to be a concept worthy of further exploration due to the increased therapeutic effect of the therapy based on simultaneous vascular targeting and its direct effect on tumor cells.

## 1. Introduction

Melanoma incidence is increasing significantly. Patients with early stages of melanoma have a good long-term prognosis compared to patients with advanced stages of melanoma. Later metastatic melanoma poses a persistent therapeutic challenge due to its aggressiveness and poor responsiveness to conventional therapies [1]. Therefore, a lot of new therapies are being developed and the introduction of immune therapy with monoclonal antibodies against immune checkpoints and targeted therapy with BRAF and MEK inhibitors [1,2,3] was a breakthrough in the treatment of advanced disease. Nevertheless, there is still a percentage of patients that do not respond to these therapies. Therefore, alternative targets are needed and one possibility for targeted therapy of melanoma is gene therapy, which is mainly used to target specific molecules that are involved in the hallmarks of cancer (e.g., RNA-based therapy for upregulated gene silencing) and DNA vaccination-based therapy for the generation of an immune response [4].

In recent years, gene electrotransfer (GET) has gained in value as a delivery system in melanoma treatment and proved to be an effective and safe method in several preclinical and clinical studies [5]. In different preclinical studies, GET has already been successfully used to target angiogenesis and the survival of melanoma cells [6,7,8,9] and also as immunotherapy with IL-12 [10,11,12], resulting in anti-tumor and anti-metastatic action. Furthermore, some promising clinical trials have already been conducted on melanoma using GET of plasmid encoding IL-12 or GET of anti-angiogenic plasmid AMEP [13,14,15].

For the targeted gene therapy approach using GET, endoglin represents a viable candidate. Endoglin is a co-receptor of transforming growth factor-β complex (TGF-β), which activates a signaling pathway resulting in increased endothelial cell proliferation, migration, and survival [16]. Therefore, it is involved in the development of tumor vasculature through promoting angiogenesis and the targeting of endoglin with antibodies resulted in an anti-tumor effect and a reduced number of blood vessels [17]. Moreover, in our recent studies, endoglin downregulation was shown to have an anti-tumor effect mediated by vascular targeted action [18,19], which was both anti-angiogenic and vascular disrupting. These studies were conducted in an endoglin non-expressing tumor model, which allowed observation of the effect of the therapy exclusively on blood vessels.

On the other hand, many human cancer cells express endoglin as its expression has been detected in different carcinoma and sarcoma cell lines [20,21,22]. Endoglin expression has also been detected in several human melanocytic lesions and cultured melanoma cells, indicating the possible involvement of endoglin in the regulation of the biological properties of melanoma cells [23,24,25]. Therefore, endoglin targeting in melanoma could benefit from an increased therapeutic effect based on simultaneously inhibiting angiogenesis and also the biological properties of melanoma cells (proliferation, migration, *etc.*). Until now only a few studies have been conducted involving melanoma and endoglin. In the study of Muñoz *et al.*, mouse B16MEL4A5 melanoma tumors were treated with anti-mouse endoglin immunotoxin and demonstrated completely and steadily blocked tumor growth after the therapy [26]. In our recent study, endoglin was silenced with plasmids encoding shRNA against endoglin in a B16F10-luc melanoma tumor model with the purpose of testing two different promoters driving shRNA expression. In this preliminary study, we already observed some effect of the therapy on the growth of melanoma tumors [27].

Therefore, to further elucidate the mechanisms behind this observation, the aim of our study was to explore the effect of endoglin silencing in melanoma on three different levels; cell cultures, 3D spheroids and tumors. We also compared the effect on two different B16 melanoma variants, one with low (B16F1) and one with high (B16F10) metastatic potential. The therapy significantly reduced the proliferation, survival and migration of melanoma cells, indicating the role of endoglin in regulating the different biological properties of melanoma cells. As similar effect was also observed also in cultured 3D melanoma spheroids. Moreover, the anti-tumor effect of the therapy was confirmed on melanoma tumors by increased tumor growth delay and tumor cures. All of these observations were confirmed in two melanoma variants with different metastatic potential in *in vitro* and *in vivo* settings, thus representing a new treatment option also for highly aggressive tumors and therefore worthy of further exploration.

## 2. Results

### 2.1. The Expression of Endoglin in Melanoma Cells and Tumors

**Figure 1:**
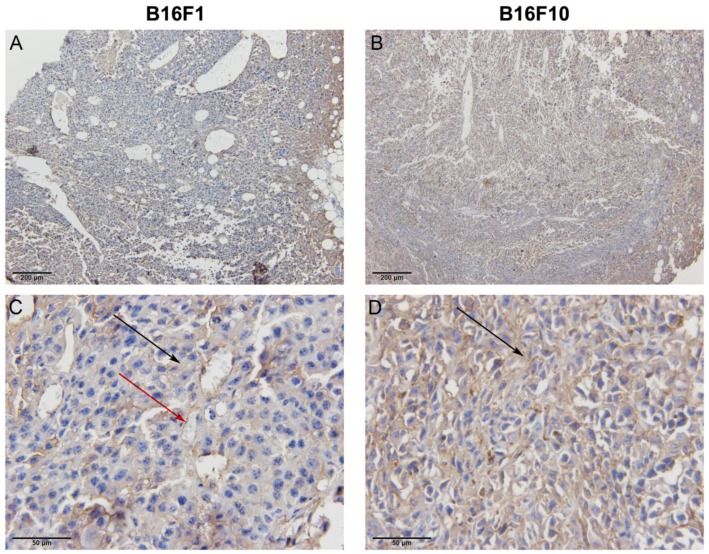
Immunohistochemical staining of endoglin (CD105) in B16F1 and B16F10 tumor models demonstrating higher expression of endoglin in the B16F10 model. Images of B16F1 (**A**) and B16F10 (**B**) tumor taken under 100× magnification and B16F1 (**C**) and B16F10 (**D**) tumor under 600× magnification. Positive brown staining is indicated with arrows, black arrows representing positive staining of the membranes and the red arrow representing positive staining of the blood vessel.

The positive effect of targeted therapies strongly depends on the expression of the target molecule. Therefore, we firstly examined the expression of endoglin in melanoma cells *in vitro* and subcutaneously induced melanoma tumors in C57Bl/6 mice *in vivo*. The expression of endoglin at the mRNA level was determined by qPCR analysis. The results demonstrated that both melanoma cell lines and tumor models express high levels of endoglin (Table 1). Delta Ct values show that B16F1 cells express 2× more endoglin than B16F10 cells and, on the contrary, B16F10 tumors express 22× more endoglin compared to B16F1 tumors. The differences are statistically significant. These results were also confirmed by immunohistochemical staining of endoglin in tumor sections of B16F1 and B16F10 tumors, where stronger staining was observed in the case of B16F10 tumors (Figure 1). Higher endoglin expression in the B16F10 tumor model could also reflect higher vascularization of B16F10 tumors, but this was not observed in our study.

**Table 1:** The expression of endoglin in melanoma cells and tumors represented as mean delta Ct ± SEM (threshold cycle) values. Delta Ct represents the difference between the Ct of endoglin and the Ct of the reference gene.

Type of Melanoma	Delta Ct (AM ± SEM)
B16F1 cells	14.2 ± 0.1
B16F10 cells	15.2 ± 0.3 *
B16F1 tumor	15.6 ± 0.2
B16F10 tumor	11.1 ± 1.4 **

AM arithmetic mean, SEM standard error of the mean; * *p* < 0.05 compared to B16F1 cells; ** *p* < 0.05 compared to B16F1 tumors.

### 2.2. The Effect of Endoglin Silencing on the Biological Properties of Melanoma Cells in Vitro

**Figure 2:**
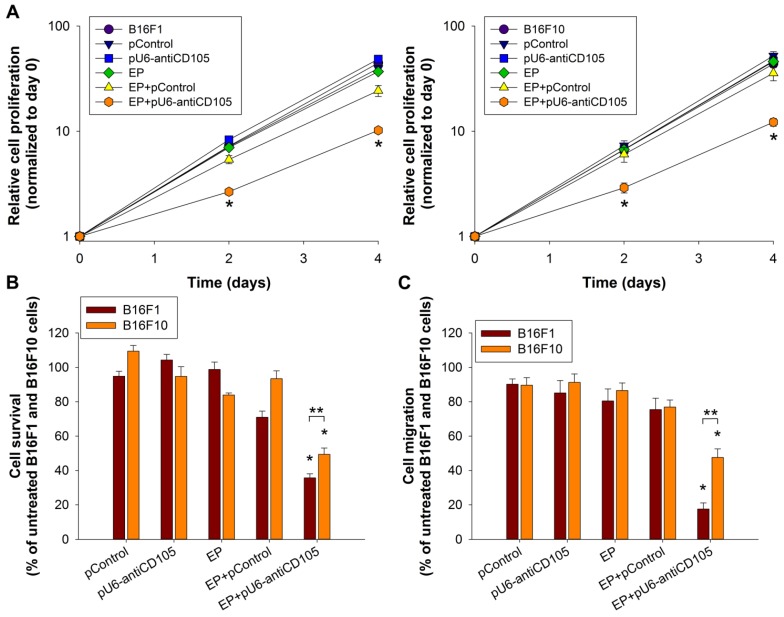
The biological properties of melanoma cells *in vitro* after endoglin silencing. (**A**) The proliferation, (**B**) survival and (**C**) migration of B16F1 and B16F10 cells after GET of pU6-antiCD105. All data for curves and bars are represented as arithmetic mean ± standard error of the mean. One asterisk indicates statistically significant differences between EP+pU6-antiCD105 and the control groups, and two asterisks between the B16F1 and B16F10 cell lines (*^,^** *p* < 0.05). Two independent experiments were performed for each assay with *n* = 8 (**A**); *n* = 3 (**B**); *n* = 4 (**C**) replicates.

Since high expression levels of endoglin in B16F1 and B16F10 cell lines were demonstrated, we examined cell proliferation, survival and migration after endoglin silencing. The proliferation of cells was monitored for four days after GET of pU6-antiCD105. Silencing of endoglin in B16F1 and B16F10 cell lines resulted in a statistically significant decrease in cell proliferation by approximately 80% and 70% (*p* < 0.05), respectively, at day 4, compared to the proliferation of untreated cells, and by 58% and 66% (*p* < 0.05), respectively compared to GET of pControl (Figure 2A). There was no significant difference between the proliferation of B16F1 and B16F10 melanoma variants after endoglin silencing. GET of pControl, the control plasmid DNA, had no statistically significant effect on the proliferation of cells.

Afterwards the survival of cells was determined by clonogenic assay after GET of pU6-antiCD105. The B16F1 and B16F10 cell colonies were formed seven days after plating. After silencing of endoglin in B16F1 and B16F10 cells the number of colonies was statistically significantly decreased by approximately 65% and 50% (*p* < 0.05), respectively, compared to the number of colonies formed from untreated cells (Figure 2B), and by approximately 50% and 47% (*p* < 0.05), respectively, compared to GET of pControl. The survival of B16F1 cells was significantly (*p* < 0.05) more reduced than that of B16F10. GET of pControl had no statistically significant effect on the survival of cells.

Since both the B16F1 and B16F10 cell lines exhibit metastatic potential, with the latter having a higher one, the effect of endoglin silencing on the migration of cells was also examined. The migration of cells was monitored for three days by an xCELLigence Real Time Cell Analyzer. Silencing of endoglin in B16F1 and B16F10 cell lines resulted in a statistically significant decrease in cell migration by approximately 80% and 50% (*p* < 0.05), respectively, compared to the migration of untreated cells, and by approximately 76% and 38% (*p* < 0.05), respectively, compared to GET of pControl (Figure 2C). The migration of B16F1 cells was significantly (*p* < 0.05) more reduced than that of B16F10. GET of the control plasmid DNA pControl had no statistically significant effect on the migration of cells.

Overall, in comparison between the melanoma B16F1 and B16F10 variants, GET of pU6-antiCD105 was more effective in the B16F1 than in the B16F10 cell line due to similar effectiveness in reducing the proliferation and survival of cells in both cell lines, whereas in B16F1 a bigger effect on reducing cell migration was observed.

### 2.3. The Effect of Endoglin Silencing on the Formation and Growth of Melanoma Spheroids

The spheroids possess intermediate complexity between 2D cell cultures *in vitro* and tumors *in vivo*, and therefore represent a valuable experimental tool for studying the complex 3D tumors’ architecture. In this study we used spheroids to examine whether melanoma cells *in vitro* are capable of forming spheroids and enabling further growth of spheroids since their biological properties were affected by GET of pU6-antiCD105.

**Figure 3:**
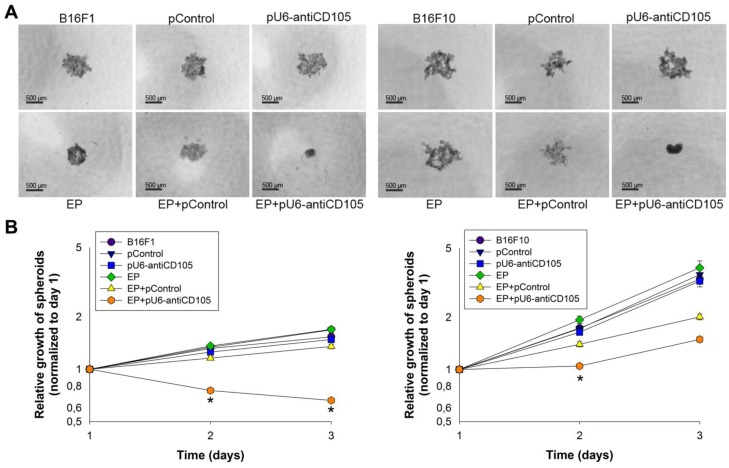
The formation and growth of melanoma spheroids after endoglin silencing. (**A**) The images of B16F1 and B16F10 spheroids 72 h after gene electrotransfer of pU6-antiCD105; (**B**) The growth of B16F1 and B16F10 spheroids after gene electrotransfer of pU6-antiCD105. All data for spheroid growth curves are expressed as arithmetic mean ± standard error of the mean. Asterisks indicate statistically significant differences between EP+pU6-antiCD105 and the control groups (* *p* < 0.05). The data are from two independent experiments with *n* = 8.

The images of the spheroids were taken 72 h after GET of pU6-antiCD105 (Figure 3A). B16F1 and B16F10 spheroids were effectively formed in all six experimental groups, although differences were noticed in their shape and size. The spheroids from the control groups (all experimental groups except EP+pU6-antiCD105) were loose, irregularly shaped, with spiky edges, and measured 999 ± 41 µm (B16F1) and 1053 ± 28 µm (B16F10) in diameter. However, the spheroids which were formed after the exposure of the cells to GET of pU6-antiCD105, appeared to be more condensed, rounded and smaller and measured 324 ± 10 µm (B16F1) and 543 ± 21 µm (B16F10) in diameter, indicating the possible increased adhesion of melanoma cells, which was previously observed also in endothelial cells [18]. All these observations relate to both B16F1 and B16F10 spheroids.

The growth of spheroids was monitored for three days after their formation. The silencing of endoglin in B16F1 and B16F10 melanoma variants resulted in a statistically significant decrease in spheroid growth by approximately 45% and 40%, respectively, at day 2 compared to the growth of spheroids that were formed from untreated cells, and 35% and 25% compared to GET of pControl (Figure 3B). The B16F1 spheroids, which were formed after GET of pU6-antiCD105, did not grow and their size was smaller every day during the measurement period, while the B16F10 spheroids started to grow from day 2 onwards.

### 2.4. The Effect of Endoglin Silencing on the Growth of Melanoma Tumors in Vivo

**Figure 4:**
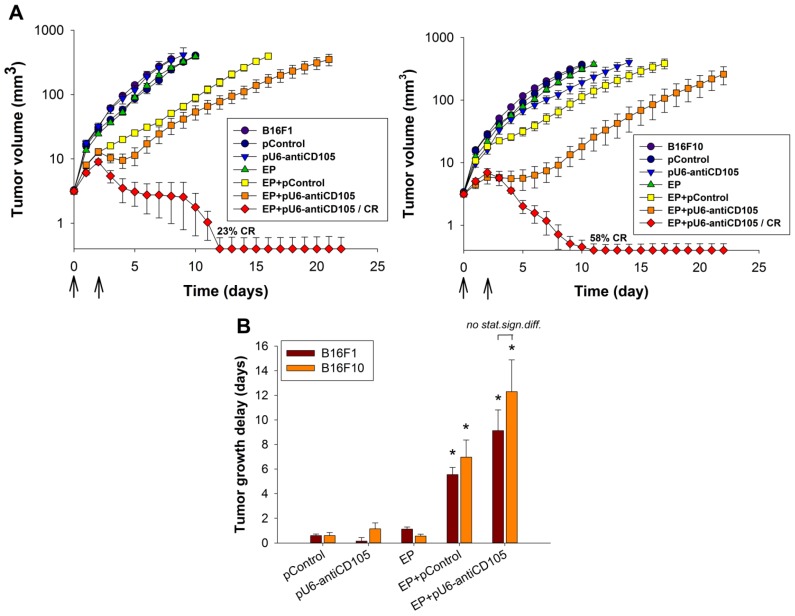
The growth of melanoma tumors in mice after endoglin silencing. (**A**) The growth of B16F1 and B16F10 tumors and their (**B**) tumor growth delay after gene electrotransfer of pU6-antiCD105. CRs were not included in the calculation. Arrows indicate the day of the treatments. All data are expressed as arithmetic mean ± standard error of the mean. Asterisks indicate statistically significant differences between EP+pU6-antiCD105 and the control groups (* *p* < 0.05). CR = complete response. The data are from one experiment with *n* = 12.

Finally, we tested the therapeutic potential of endoglin silencing in subcutaneously induced melanoma tumors in mice. The treatment of melanoma tumors with GET of pU6-antiCD105 resulted in tumor growth delay and tumor cures (mice remained tumor-free for 100 days) (Figure 4). The anti-tumor effect in B16F1 and B16F10 tumors was statistically significant, producing 9.1 ± 1.7 and 12.3 ± 2.6 days tumor growth delay and in 23% and 58% complete responses, respectively, compared to untreated tumors. GET of the control plasmid pControl also resulted in significant tumor growth delay, by 5.6 ± 0.6 days in B16F1 and 7.0 ± 1.4 days in B16F10 tumors. This was already observed in previous studies that electrotransfer of DNA can induce regression of B16 tumors, as tumor regression was observed after delivery of single-stranded or double-stranded DNA in both immunocompetent and immunodeficient mice under different pulsing protocols [28,29].

## 3. Discussion

In this study we demonstrate that the targeting of endoglin with GET of plasmid DNA encoding shRNA against endoglin (pU6-antiCD105) represents a new option for targeted therapy of melanoma. The therapy significantly reduced the proliferation, survival and migration of melanoma cells, indicating the role of endoglin in regulating the different cellular properties of melanoma cells. This was observed also in cultured 3D melanoma spheroids, which were formed from treated cells, as the therapy resulted in the reduced growth of spheroids and also in the altered shape of spheroids, which were more condensed, rounded and smaller, indicating the possible increased adhesion of melanoma cells. Moreover, the anti-tumor effect of the therapy was confirmed in melanoma tumors by increased tumor growth delay and tumor cures. All of these observations were confirmed in two melanoma variants with different metastatic potential in *in vitro* and *in vivo* settings, thus representing a new treatment option also for highly aggressive tumors that is worthy further exploration.

Based on various studies, endoglin proved to be a valid target for anti-angiogenic therapy [16,30]. In the studies using antibodies against endoglin, significant tumor growth delay and anti-metastatic action was achieved [17,31], resulting already in phase I clinical trials for the treatment of cancer with the TRC105 anti-endoglin antibody [16,32,33]. Another approach was used in our group, using GET of plasmid DNA encoding shRNA against endoglin (pU6-antiCD105), which was demonstrated to be effective in the knockdown of endoglin on endothelial cells *in vitro* (approximately 50% knockdown) and the B16F10-luc melanoma model *in vivo* (approximately 80% knockdown) [18,27]. When transfected into the tumors using GET, the therapy demonstrated both anti-angiogenic and vascular disrupting action [18]. In contrast to several studies exploring endoglin in terms of endothelial cells and angiogenesis, little is known about the role of endoglin in cancer cells, although the expression of endoglin has already been detected in cancer cells of different origin [22,24]. In our study, we confirmed that endoglin is expressed in two melanoma variants, B16F1 and B16F10. The expression of endoglin was higher in the B16F1 cell line in the case of cell cultures; however in induced tumors in mice, B16F10 tumors had a higher expression of endoglin, which was confirmed by qPCR and immunohistochemical staining.

To the best of our knowledge, our study is the first exploring the effect of endoglin on the biological properties of two different metastatic melanoma variants. We confirmed on cell cultures and 3D spheroids that silencing of endoglin with GET of pU6-antiCD105 plasmid alters the biological properties of both B16F1 and B16F10 melanoma. The survival, proliferation and migration of cells were reduced after the therapy and accordingly also slower growth and a more compact shape of spheroids was observed. These outcomes are consistent with our previous studies, where increased adhesion and reduced proliferation and migration were also observed in endothelial cells [18] and the reduced number of metastases following endoglin silencing was demonstrated in the melanoma model [27]. Although significant changes in biological properties were observed in both melanoma variants, the effect was more pronounced in B16F1, probably due to a higher expression level of endoglin.

A significant anti-tumor effect was also confirmed in *in vivo* experiments on subcutaneous melanoma tumors B16F1 and B16F10. In contrast to *in vitro* experiments, the B16F10 melanoma variant responded better to the therapy with GET of pU6-antiCD105 plasmid due to a higher expression of endoglin, resulting also in a high percentage of tumor cures. Higher endoglin expression in the B16F10 tumor model could also reflect higher vascularization of B16F10 tumors and this could further enhance the anti-tumor effect in B16F10 tumors. Such a pronounced anti-tumor effect on both melanoma variants was not expected based on the previous study with GET of the same plasmid in B16F10-luc melanoma cells, which resulted in shorter tumor growth delay and also no tumor cures being achieved [27]. The reason for the obtained major differences is currently not known, but it could be attributed to higher amount of plasmid DNA used in the current study. Another factor is the tumor model, as we used a transgenic cell line in our preliminary study, stably expressing luciferase. This transgenic cell line expresses higher levels of endoglin (delta Ct 9.7) and this could attribute to poorer response to the therapy after endoglin silencing.

In our previous study, in an endoglin non-expressing TS/A adenocarcinoma tumor model, the anti-tumor effect of pU6-antiCD105 plasmid was evaluated with the aim of demonstrating the anti-vascular effect of endoglin silencing [18]. In that tumor model, we demonstrated the anti-vascular effect of endoglin silencing, which was composed of an anti-angiogenic as well as a vascular disrupting action. The therapy resulted in prolonged tumor growth delay, but no tumor cures were achieved, whereas in this study on endoglin expressing B16 melanoma tumors, a high percentage of tumor cures and longer growth delay of tumors were achieved. The results of our study clearly demonstrate the dual mechanism of action of the anti-endoglin therapy on blood vessels as well as on melanoma cells.

In conclusion, endoglin targeting for the treatment of melanoma seems to be a concept worthy of further exploration due to the increased therapeutic effect of the therapy based on the simultaneous vascular targeted effect as well as the direct effect on tumor cells. Melanoma treatment has improved substantially over the last few years due to the development of immunological treatments, however a certain percentage of patients are unresponsive to these treatments [34]. Therefore, further studies on our promising treatment approach are needed in this field, in terms of its combination with other treatment modalities and also by elucidating the molecular mechanisms contributing to the anti-tumor effect.

## 4. Experimental Section

### 4.1. Cell Lines

Murine melanoma cell lines, B16F1 with low and B16F10 with high metastatic potential (American Type Culture Collection, Manassas, VA, USA), were cultured in an advanced minimum essential medium (AMEM; Gibco, Thermo Fisher Scientific, Waltham, MA, USA), supplemented with 5% fetal bovine serum (FBS, Gibco), 10 mL/L L-glutamine (GlutaMAX, Gibco), 100 U/mL penicillin (Grünenthal, Aachen, Germany) and 50 µg/mL gentamicin (Krka, Novo mesto, Slovenia) in a 5% CO_2_ humidified incubator at 37 °C.

### 4.2. Determination of Endoglin Expression with qPCR Analysis

To determine the expression of endoglin at the mRNA level in B16F1 and B16F10 cells *in vitro* and tumors *in vivo*, total RNA extraction and qPCR analysis were performed. For *in vitro* determination, cells were trypsinized and a cell suspension was prepared and centrifuged. Thereafter, total RNA was extracted from the cells. For *in vivo* determination, 2 to 4 tumors per tumor model were collected from euthanized mice and the tumors were immediately frozen in liquid nitrogen. Thereafter, the frozen tumors were homogenized in a mortar with a pestle and the total RNA was extracted with TRIzol Plus RNA Purification Kit (Invitrogen, Thermo Fisher Scientific) according to the manufacturer’s protocol. The RNA concentration was quantified by a spectrophotometer at 260 nm (Epoch, Biotek, Winooski, VT, USA) and the purity of the RNA was quantified by determining the ratio of absorbance at 260/280 nm and 260/230 nm. A SuperScript VILO cDNA Synthesis Kit (Invitrogen, Thermo Fisher Scientific) was used for reverse transcription of one µg of total RNA according to the manufacturer’s instructions. Ten times diluted mixtures were used as a template for quantitative PCR using TaqMan Gene Expression Assay (Applied Biosystems, Thermo Fisher Scientific) and TaqMan Gene Expression Master Mix (Applied Biosystems). The Taqman Gene Expression Assay contained a pair of primers and TaqMan probes for the amplification of the fragment of mouse endoglin cDNA (Mm00468256_m1). For internal control, murine 18S ribosomal RNA (Mm03928990_g1) was used. Quantitative PCR was performed on a 7300 System (Applied Biosystems) as follows: 2 min at 50 °C, 10 min at 95 °C, 45 cycles of 15 s at 95 °C and 1 min at 60 °C. Threshold cycle values (Ct) of endoglin and 18S were determined with 7300 System SDS software (Applied Biosystems). The delta Ct value was later determined as the difference between the Ct of endoglin and the Ct of 18S. Delta Ct values signify the expression of endoglin and lower values represent a higher expression. The comparison between B16F1 and B16F10 melanoma variants was made by calculating the difference between delta Ct values (x) and calculating $2^{x}$.

### 4.3. Plasmids

Two plasmids were used for gene electrotransfer: the therapeutic plasmid pU6-antiCD105 encoding shRNA against endoglin under the control of constitutive U6 promoter [18] and the blank plasmid pControl under the control of constitutive CMV promoter [6]. The therapeutic plasmid pU6-antiCD105 targets both L- and S- endoglin isoforms. The blank plasmid pControl was of similar size to the therapeutic plasmid (approximately 3500 base pairs) and contained only a backbone without any coding sequence. Amplification of both plasmids was performed in a competent *E. coli* (TOP10; Invitrogen, Thermo Fisher Scientific) and purified using a Qiagen Maxi-Endo-Free Kit (Qiagen, Hilden, Germany) according to the manufacturer’s protocol. The quality and quantity of the isolated plasmid DNA were determined using a spectrophotometer (Epoch Microplate Spectrophotometer, Take3™ Micro-Volume Plate, BioTek) and agarose gel electrophoresis (Scie-Plas Ltd, Cambridge, UK). A working concentration of 1 mg/mL (for *in vitro* experiments) or 4 mg/mL (for *in vivo* experiments) was prepared with endotoxin-free water.

### 4.4. In vitro Gene Electrotransfer (GET)

The B16F1 or B16F10 cells grown as a monolayer were trypsinized and collected in a cell suspension in an ice-cold buffer (125 mM sucrose, 10 mM K_2_HPO_4_, 2.5 mM KH_2_PO_4_, 2 mM MgCl_2_x6H_2_0). For GET, a dense cell suspension with a concentration of 25 × 10^6^ cells/mL was prepared. To 44 µL of prepared cell suspension 11 µL of plasmid DNA was added (11 µg). Then 50 µL of the resulting mixture (1 × 10^6^ cells and 10 µg of plasmid) was pipetted between two parallel stainless-steel plate electrodes with a 2 mm gap, connected to an electric pulse generator GT-01 (Faculty of Electrical Engineering, University of Ljubljana, Ljubljana, Slovenia) and subjected to eight square-wave electric pulses with a voltage-to-distance ratio of 600 V/cm, pulse duration of 5 ms and repetition frequency of 1 Hz. After the application of electric pulses, the cells were incubated for 5 min with 100 µL of FBS and then plated in the AMEM culture medium for further proliferation, clonogenic and migration assay, and 3D spheroid formation.

In addition to the GET of therapeutic plasmid DNA (EP+pU6-antiCD105), there were also five control groups: untreated cells (B16F1 or B16F10), cells treated with therapeutic or control plasmid DNA only (pU6-antiCD105 or pControl), cells exposed to electric pulses without plasmid DNA (EP) and GET of the control plasmid (EP + pControl).

### 4.5. Cell Proliferation Assay

To determine the effect of endoglin silencing on cell proliferation, 2 × 10^2^ B16F1 or B16F10 cells per well were plated 24 h after GET of pU6-antiCD105 on 96-well plates (Corning Inc., Corning, NY, USA) in 100 µL of AMEM culture medium, containing FBS and antibiotics. The cells were incubated at 37 °C in a 5% CO_2_ humidified incubator. Cell viability was measured at day 0, 2 and 4 with a Presto Blue assay (Invitrogen, Thermo Fisher Scientific). Ten µL of Presto Blue reagent were added to each well and after 1.5 h of incubation at 37 °C in a 5% CO_2_ humidified incubator the fluorescence intensity (Ex = 535–560 nm, Em = 590–615 nm) was measured with a microplate reader (Infinite 200, Tecan, Männedorf, Switzerland). The proliferation curve of each experimental group was normalized to day 0.

### 4.6. Clonogenic Assay

To determine the effect of endoglin silencing on the survival of B16F1 and B16F10 cells 24 h after GET of pU6-antiCD105, the cells (200 or 300 cells/petri dish) were plated in petri dishes with 4 mL of AMEM culture medium. When the cell colonies were formed, they were fixed and stained with a crystal violet solution (Sigma-Aldrich, St. Louis, MO, USA) and counted. The colonies containing less than 50 cells were disregarded. The cell survival for each experimental group was normalized to the survival of untreated cells.

### 4.7. Migration Assay

To determine the effect of endoglin silencing on the migratory potential of B16F1 and B16F10 cells 24 h after GET of pU6-antiCD105, an xCELLigence Real Time Cell Analyzer (RTCA; Roche Diagnostics GmbH, Mannheim, Germany) and CIM-plate 16 wells (Roche) were used. The bottoms of the CIM-plates were coated with 0.3 µg of human fibronectin (BD Biosciences, San Jose, CA, USA) and incubated in a laminar air flow chamber for 30 min. The upper compartments of the CIM-plates were coated with 0.5 μg of human fibronectin (BD Biosciences) and incubated for 2 h at 37 °C in 5% CO_2_. Afterwards, the upper compartments were washed with 50 µL of phosphate-buffered saline (PBS, Merck Millipore, Darmstadt, Germany). The lower compartments were filled with 180 μL of AMEM culture medium containing FBS as a chemoattractant. The top and bottom compartments of the CIM-plates were then assembled together and 80 μL of AMEM culture medium without FBS was added to the top compartment. The assembled CIM-plates were allowed to equilibrate for 10 min at 37 °C and 5% CO_2_ prior to the addition of the cells. The suspension of B16F1 or B16F10 cells (1 × 10^4^ cells in 80 µL of FBS-free AMEM culture medium) was plated into the top chambers of the CIM-plates and placed into the xCELLigence system for data collection. Impendence data, reported as the cell index, were collected with the xCELLigence software every 15 min during the following 72 h. The migration of the cells was shown as a curve in a two-dimensional system (time, cell index). For the analysis of the data, only the linear part of the curve was considered. In the interval where the curves were linear, the slopes of the curves were compared and the percentage of migration (%) was calculated by the ratio of the slope of the treated cells to the slope of the untreated control cells.

### 4.8. 3D Spheroid Cultures

In order to examine the effect of endoglin silencing on the formation and growth of B16F1 and B16F10 spheroids, 3D cell culture models-spheroids were formed 24 h after GET of pU6-antiCD105. The suspension of B16F1 or B16F10 cells (1 × 10^3^ cells in 150 µL of AMEM culture medium with FBS and antibiotics) was plated into a round-bottomed 96-well ultra-low attachment microplate (96 Well Clear Round Bottom Ultra Low Attachment Microplate, Corning) using a manual multi-channel pipette. The 96-plate was then centrifuged at 1500 rpm and room temperature for 3 min, and then gently placed in the humidified incubator with 37 °C and 5% CO_2_. The spheroids were formed already 24 h after seeding. For the optimal growth of spheroids the AMEM culture medium was changed every second day.

### 4.9. Spheroid Growth

The growth of B16F1 and B16F10 spheroids was monitored by capturing the images with a digital camera (DP72, Olympus, Hamburg, Germany) connected to an inverted microscope (IX70, Olympus) every day. The images of the spheroids were further analyzed at all-time points with CellSens Dimension software (Olympus), which allows the measurements of their diameter and surface area. The spheroid growth curve of each experimental group was normalized to day 1 (formation of spheroids).

### 4.10. Animal Models and Tumor Induction

Female C57Bl/6 mice, 6–8 weeks old, purchased from Envigo (Udine, Italy) were used in the experiments. For housing the mice, specific pathogen-free conditions were used, the temperature was maintained at 20–24 °C, the relative humidity at 55% ± 10% and a 12 h light/dark cycle was provided. Water and food were provided *ad libitum*. The animals were adapted to their new environment for 2 weeks before the experiments were conducted. All procedures were approved by the Veterinary Administration of the Ministry of Agriculture, Forestry and Food of the Republic of Slovenia (permission No. 34401-2/2012/4, date of approval 3/2/2012) and performed in compliance with the guidelines for animal experiments of the EU directive (2010/63/EU) and according to the standards stated in the eighth edition of the Guide for the Care and Use of Laboratory Animals [35]. Animal protocols were in accordance with FELASA guidelines and the National Law for Laboratory Animal Experimentation (Law No. 18.611). For the induction of subcutaneous B16F1 and B16F10 tumors, a suspension of 1 × 10^6^ B16F1 or B16F10 cells prepared from a cell culture *in vitro* in 100 µL of physiological solution was injected into the shaved right flank of the mice. When the tumors reached approximately 3 mm in the largest diameter (2–3 days after subcutaneous injection of cells), the animals were randomly divided into 6 experimental groups, consisting of 12 animals per group and subjected to a specific experimental protocol.

### 4.11. In Vivo Gene Electrotransfer (GET)

*In vivo* GET of pU6-antiCD105 into B16F1 or B16F10 subcutaneous tumors was performed 2 times on day 0 and 2 with the injection of 100 µg of plasmid DNA in 25 µL of endotoxin-free water.

The first experimental group (Control) of animals was injected intratumorally (i.t.) with 25 µL of endotoxin-free water. The second (pControl) and third groups (pU6-antiCD105) of animals were injected i.t. with 100 µg of pControl or pU6-antiCD105 in 25 µL of endotoxin-free water. The fourth group (EP) of animals was injected i.t with 25 µL of endotoxin-free water. The fifth (EP+pControl) and sixth groups (EP+pU6-antiCD105) of animals were injected i.t. with 100 µg of pControl or pU6-antiCD105 in 25 µL of endotoxin-free water. The animals were kept under inhalation anesthesia with 1.5% isoflurane (Izofluran Torrex para 250 mL, Chiesi Slovenia, Ljubljana, Slovenia) and an oxygen flow of 1 l/min during the procedure.

Electric pulses (2 sets of 4 pulses in perpendicular directions at a frequency of 1 Hz, amplitude over distance ratio of 600 V/cm and 5 ms duration time) were delivered 10 min after i.t. injection to the tumors of the fourth, fifth and sixth groups through 2 parallel stainless steel electrodes with a 4 or 6 mm distance between them. A water-based gel (Ultragel, Budapest, Hungary) was used to ensure good conductivity in the contact of the electrodes with the skin overlying tumors. Electric pulses were generated by the electric pulse generator ELECTRO CELL B10 (LEROY biotech, Betatech, Saint-Orens-de-Gameville, France).

### 4.12. Tumor Growth

To determine the effect of endoglin silencing on the growth of subcutaneous B16F1 or B16F10 tumors, tumors were measured every second day after the first treatment in 3 orthogonal diameters with a Vernier caliper, and their volumes calculated using the equation V = a × b × c × π/6. Tumor growth was monitored until the tumors reached between 300 and 350 mm^3^, thereafter the animals were euthanized. Mice with fully regressed primary tumors that remained tumor-free for 100 days were termed as cured (complete response) and were thereafter euthanized. The tumor growth delay for each experimental group was calculated as the difference in time when treated and untreated tumors reached a volume of 40 mm^3^, and the mice with complete response were excluded from the calculation. Animal weight was measured every second day and was used as a general index of systemic toxicity.

### 4.13. Histology

For the evaluation of endoglin expression *in vivo*, immunohistochemical staining of endoglin was performed. From the control groups, two B16F1 and B16F10 bearing mice were sacrificed; tumors were excised, fixed in IHC zinc fixative (BD Pharmingen, BD Biosciences) and embedded in paraffin. Two µm thick sections were cut from each paraffin block and used for immunohistochemical staining of endoglin (CD105). Sections were incubated with primary goat polyclonal antibodies against endoglin (AF1320, R&D Systems, Minneapolis, MN, USA) at a dilution of 1:1800. A peroxidase-conjugated streptavidin-biotin system (Anti-Goat HRP-DAB Cell & Tissue Staining Kit, R&D Systems) was used as the colorogenic reagent followed by hematoxylin counterstaining.

The immunohistochemically-stained slides were observed under light microscopy at room temperature and from each slide two images at different magnifications were captured with a DP72 CCD camera (Olympus) connected to a BX-51 microscope (Olympus).

### 4.14. Statistical Analysis

SigmaPlot Software (version 12.0, Systat Software, London, UK) was used for the statistical analysis and graphical representation. All data were tested beforehand for normality of distribution using the Shapiro-Wilk test. All quantitative data are presented as arithmetic mean (AM) ± standard error (SEM). The letter n denotes the number of replicates in each experiment. The differences between the experimental groups were statistically evaluated by One Way ANOVA analysis of variance followed by the Holm-Sidak test for multiple comparisons. A p-value of less than 0.05 was considered to be statistically significant.
